# Development and validity of the Value-based Stigma Inventory (VASI): a value-sensitive questionnaire for the assessment of mental health stigma

**DOI:** 10.1186/s12888-021-03427-4

**Published:** 2021-11-15

**Authors:** Sophia Rieckhof, Christian Sander, Sven Speerforck, Elke Prestin, Matthias C. Angermeyer, Georg Schomerus

**Affiliations:** 1 Department of Psychiatry and Psychotherapy, University of Leipzig Medical Center, Semmelweisstrasse 10, D-04103 Leipzig, Germany; 2 Department of Psychiatry II, University of Ulm, Ulm, Germany; 3 Center for Public Mental Health, Gösing am Wagram, Austria

**Keywords:** Stigma, Mental illness, Values, Scale development

## Abstract

**Background:**

It has been hypothesized that mental illness stigma differs according to what matters most to people, and that this results in value-based differences in stigma within societies. However, there is a lack of stigma measures that account for a broad range of values, including modern and liberal values.

**Methods:**

For the development of the Value-based Stigma Inventory (VASI) a preliminary item-pool of 68 VASI-items was assembled by mental health and stigma experts. For psychometric evaluation, we tested the VASI in an online sample of the general population (*n* = 4983).

**Results:**

Based on item-characteristics as well as explorative and confirmatory factor analyses, a final version of the VASI was developed, comprising 15 items and 5 subscales. The VASI shows good psychometric properties (item difficulty = 0.34 to 0.67; mean inter-item correlation *r =* 0.326; Cronbach’s α = 0.879). Medium to high correlations with established stigma scales (SDS, SSMI), medium associations with instruments assessing personal values (PVQ, KSA-3) and small to no associations with a social desirability scale (KSE-G) attest to good convergent and discriminatory validity of the new instrument. Normative values for the VASI subscales are presented.

**Conclusions:**

The developed VASI can be used to assess public stigma of mental illness including personal stigma-relevant value orientations.

**Supplementary Information:**

The online version contains supplementary material available at 10.1186/s12888-021-03427-4.

## Background

The stigma of mental illness has been shown to vary between different cultures [1]. To conceptualize cultural differences in mental illness stigma, Yang and co-workers [2] have introduced the theory of *What Matters Most* (WMM), predicting that stigma is most visible when threatening the loss or diminution of what matters most within a cultural context. They argue that culture specific measures of stigma are needed to measure stigma in those cultural interactions that are most relevant to the respondents of a specific cultural background.

Not only between, but also within cultures there are differences of WMM, visible for example in the growing polarization of Western societies. Inglehart and Norris [3] found that it is not so much socioeconomic status but primarily opposing cultural world views that play a central role in the formation of the Left-Right cleavage. They state that the shift to post-materialistic and cosmopolitan values is accompanied by a protesting departure from liberal values of tolerance and self-realization. Differentiations between the value dimension of the progressive, cosmopolitan milieu and the traditionalist, communitarian milieu are made by various authors [4–6].

For understanding such differences in mental illness stigma within cultures, Schomerus and Angermeyer [7] apply the WMM theory to the formation of stigma within Western societies. The authors hypothesize that, although conservative/authoritarian values have traditionally been found to be associated with mental illness stigma, liberal/modern values can similarly lead to stigma, but have not been thoroughly examined in this respect. Assuming a ‘blind spot in stigma research’, Schomerus and Angermeyer [7] hypothesize that liberal-minded people might also stigmatize people with mental illness, if these are perceived as a potential threat to their liberal values.

The theory that individuals of various outgroups are more likely prejudiced and discriminated if they are perceived as a threat to personal values is empirically supported [2, 8–13]. Brandt and coworkers [14] showed in three independent studies intolerance of both conservatives and liberals of groups whose values and worldviews contradict with their ideological worldview. To counter the assumed blind spot among mental illness stigma research, instruments are needed that measure mental illness stigma with regard to situations relevant both to people with conservative/authoritarian and liberal/modern values.

### Aims & research hypotheses

The present study reports on the development and validation of a Value-based Stigma Inventory (VASI). To ensure the validity of the instrument, we examine aspects of discriminatory and convergent validity, setting up several assumptions. According to Cohen [15], correlation coefficients between the new instrument and established stigma scales should be moderate to high (≥0.5) and moderate (> 0.3) between the new instrument and established value scales. Both authoritarian and liberal values should be associated with increased stigmatization in respective subscales. Finally, correlation coefficients between the new instrument and an established social desirability scale should be small.

## Methods

### Creation of a preliminary item pool (VASI-68)

First, based on the heuristic model of party competition in Western societies by Inglehart and Norris [3] and the theory of basic values of Schwartz [16] we distinguished six possible value orientations: liberal, authoritarian, community welfare, individual well-being, performance and hedonism. Second, items were generated by a team of mental health stigma experts and a person with lived experience (MA, EP, SR, GS) relating stigma and exclusion to potential conflicts with these value orientations. For example, the item *If you are living with a person with mental illness, it is difficult to lead a life according to your own ideas* was created to illustrate a potential conflict with individualism and hedonism. Third, by means of a rating by an extended team of experts (including MA, SR, CS, SS and GS), items were reformulated and reduced from 156 to 68, taking into account criteria such as comprehensibility, unambiguity, everyday relevance, face validity and possible response tendencies.

### Data collection

A sample of the German adult population who were registered in a professional online-access panel (KANTAR) responded to an online survey containing 68 VASI items and other questionnaires related to mental health stigma and personal values. The study was approved by the Ethics Committee of the University of Leipzig (239/20-ek) and conducted in accordance to the declaration of Helsinki. All participants provided informed consent. Data collection was carried out in cooperation with a social and market research institute (USUMA).

Sampling was stratified for age, gender and place of residence of the respondents, resulting in a sample matching the general population for these characteristics. A total of 8136 people responded to the invitation to the survey. Of these, 199 (2.4%) did not agree to participate, 681 (8.4%) did not complete the questionnaire and 754 (9.3%) could not participate since the predefined quota for age, gender or place of residence were already accomplished.

After pretesting the questionnaire, a minimum time of 11 min to respond to all items had been determined to reduce the number of rapid guessers [17]. A total of 1.480 (18.2%) responders fell below this time limit and were therefore excluded. A further 10 data sets had to be excluded due to a high proportion of item non-response. In total, 5012 unique datasets were obtained.

### Sample

From the dataset of 5012 cases, 29 datasets (0.6%) were excluded due to missing values in at least one of the 68 VASI items. Therefore, the final sample comprises of 4983 data sets. Of these, 2373 (47.6%) were from male and 2604 (52.3%) from female participants, while 6 participants (0.1%) identified as divers. The average age of the sample was 47.4 years (SD = 14.387, range 18-74 years). Further socio-demographic data can be found in Table 1.

**Table 1.** Characteristics of the study population

Characteristics	German Population ^a^	Total Sample (*n* = 983)
Gender, n (%)		
male	48.9	47.6
female	51.1	52.3
divers	–	0.1
Age-Groups, n (%)		
18–24 years	9.0	8.9
25–34 years	15.2	14.1
35–44 years	14.7	17.1
45–54 years	17.2	22.7
55–64 years	17.8	24.1
≥ 65 years	26.0	13.0
Educational Level, n (%)		
low	33.6	12.5
middle	29.9	37.5
high	32.5	50.0

^a^Annotation: population data according to the Federal Statistical Office (www-genesis.destatis.de): data on gender and age groups as of 31 December 2019 based on extrapolation of the population trend after the 2011 microcensus; data on educational level based on the 2018 survey of the population > 15 years (excluding the population in shared accommodation)

### Survey

The survey was subdivided in order to allow the use of different instruments, while at the same time ensuring a tolerable survey length for the individual participant. All survey participants first answered the preliminary VASI items. The items were presented in randomized order to avoid sequence effects. Participants were asked to respond to each item on a 5-point Likert scale ranging from 1 (strongly disagree) to 5 (strongly agree).

**Fig. 1:**
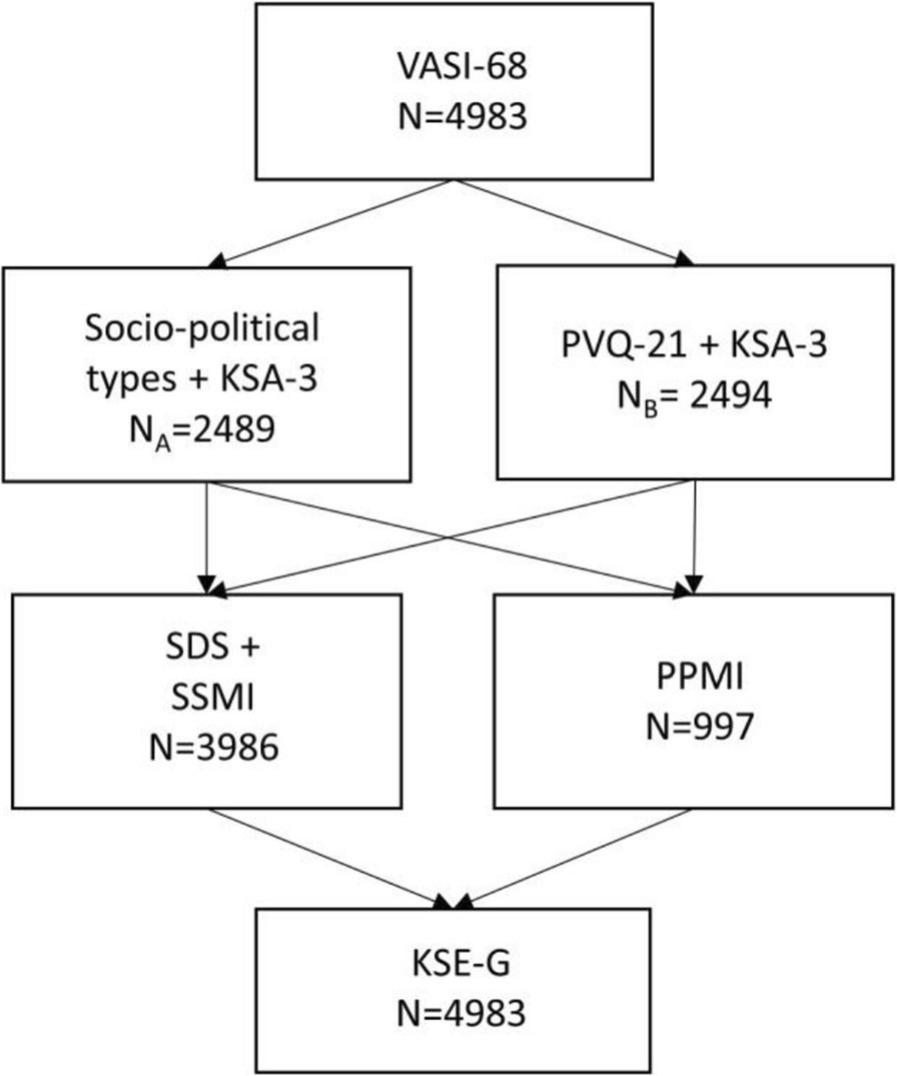
Survey structure

After answering the VASI-items, participants were randomly assigned to one of two groups differing with respect to the applied value measures (see Fig. 1). Subgroup A (*N* = 2489) processed several items on socio-political opinions that are not used for this publication. Subgroup B (*N* = 2494) answered the Portrait Values Questionnaire (PVQ, [18]). The 21-item version (PVQ-21) was used as applied in the European Social Survey (ESS). For a better representation of the subscale universalism, an additional item from the long version representing the subscale *universalism* was added (*He wants everyone to be treated justly, even people he doesn’t know. It is important to him to protect the weak in society*, [19]). Both subgroups answered the Authoritarianism Short Scale (KSA-3 [20]).

Afterwards, both groups were again divided randomly (ratio 4:1) into two subgroups, which answered different stigma scales. The first subgroup (*N* = 3986) processed the subscales *Aware* and *Agree* of the Self-Stigma in People with Mental Illness scale (SSMI-short form [21], German version: [22]) and the Social Distance Scale (SDS [23], German version: [24]). The latter was used in a modified version with two additional items for a more contemporary recording of social distance (*To what extent would you invite such a person to your home?, To what extent would you give such a person your phone number?*). The second subgroup (*N* = 997) answered the Prejudice against people with mental Illness scale (PPMI [25], results not used for this publication). Finally, all participants completed the Social Desirability–Gamma Short Scale (KSE-G [26]).

### Statistical analyses

For psychometric analysis, item distributions were tested for normality using the Shapiro-Wilks test. Item difficulty was calculated as pm = sum of item scores / (N * maximum item score) and item-total correlations (r_it_) were calculated as measure of item discriminatory power. According to Bortz and Döring [27] discriminatory power should be > 0.5, while items with discriminatory power < 0.3 therefore require a scale revision, e.g. by deleting the affected items.

Exploratory and confirmatory analyses of the factorial structure were carried out in two independent subgroups to which the survey participants were randomly assigned during the study. First, in subsample A exploratory factor analyses were carried out using a principal component analysis (PCA) approach, including all items of the preliminary VASI item pool with satisfactory item discriminatory power (r_it_ > 0.3). The Kaiser-Meyer-Olkin (KMO) measure and the Bartlett test were used to check if requirements for factor analysis were met. Eigenvalues (> 1) and the scree plot are used as criteria for determining the optimal number of factors. To obtain factors that are as uncorrelated as possible, an orthogonal rotation technique was used. According to Gorsuch [28], a varimax rotation is inappropriate because a general factor (mental health stigma) can be expected. Therefore, an Equamax rotation was chosen, which would distribute the variance of the factor loadings as evenly as possible over the factors. Factor loadings < 0.3 were not considered, as such low factor loadings indicate that a variable has less than 10% common variance with the factor [29, 30].

Subsequently, confirmatory factor analyses (CFA) using maximum likelihood estimations were performed in subsample B to confirm the factorial structure. Normality of the data was examined using the Mardia test. The adequacy of the model fit was assessed using goodness-of-fit indices recommended by Beauducel and Wittmann [31] and DiStefano and Hess [32], with cutoffs specified in various publications [33–35]: Chi^2^-test (with Bollen-Stine-Bootstrap in case of multivariate non-normality); Comparative Fit Index (CFI, cutoff ≥0.95), Tucker-Lewis Index (TLI, cutoff > 0.95), Root Mean Square Error of Approximation (RMSEA, cutoff < 0.06) and Standardized Root Mean Residual (SRMR, cutoff < 0.08).

Scores of the resulting VASI subscales were analysed using descriptive statistics: mean, standard deviations as well as quartiles and selected percentiles. Cronbach’s α was computed as a measure of internal consistency. To investigate discriminative and convergent validity of the VASI, Spearman’s rank correlations coefficients were calculated.

The significance level was set at α = 0.05 (two-tailed). All statistical analyses were conducted using the statistical software SPSS 24 (IBM SPSS Statistics for Windows, version 24). For the confirmatory factor analyses, IBM SPSS AMOS 26.0 was used.

## Results

Table 1 depicts the sociodemographic characteristics of the total sample (compared to the German population). The overall sample corresponds to the German population in terms of gender distribution, but comprises fewer participants in the age segment > 65 years, as these population strata can generally hardly be reached by online surveys. With regard to the educational level, our sample shows a higher proportion of respondents with high educational levels, which is also typical of online studies. The two subgroups A and B were created by random assignment and accordingly do not differ in terms of the socio-demographic characteristics.

### VASI-68

#### Item analysis

The 68 preliminary VASI-items were first subjected to an item analysis (results not shown). The Shapiro-Wilks test confirmed that the distribution of all items differed significantly from a normal distribution (all *p* < .01). Out of the 68 items, 53 showed a significantly positive skewness (maximum 1.47), while 10 items were negatively skewed (minimum − 0.42). Kurtosis was significantly negative in 53 items (minimum − 0.93) and positive in 11 items (maximum 1.65). Difficulty indices were of medium size (range 0.34 to 0.84). The corrected item-total-correlations (r_it_) ranged from 0.11 to 0.74 with r_it_ < 0.3 in 6 items, which were therefore removed from the VASI item pool for the next evaluations.

#### Exploratory factor analysis and item reduction

In order to determine the factorial structure of the VASI and to reduce the number of items, an explorative factor analysis was carried out on the remaining 62 VASI items in subsample A (*N* = 2989). The Kaiser-Meyer-Olkin (KMO) measure of 0.980 and the Bartlett test (*p* < .001) showed that the requirements for factor analysis were met. A Principal Component Analysis (PCA) resulted in seven components with eigenvalues (EV) > 1, explaining a cumulative 53.3% of the variance. Based on the screeplot a strong general factor (EV = 21.018, explained variance 33.9%) could be identified on which all items had factor loadings > 0.3 with polarities corresponding to the direction of the respective item wording.

Since item compilation had been based on six topics and the EV of the 7th component (1.001) was only slightly above the usual cutoff, in the next step a PCA with Equamax-rotation was performed with a fixed amount of six factors, which still explained a large amount of variance (51.6%). All items had factor loadings >.300 on at least one component, yet several items showed cross loadings > 0.3 on at least one additional component.

Based on the item loadings, components could be described meaningfully in terms of content, although some deviations from the original six topics of the item compilation were noted. In the sense of the intended item reduction, for each component, the three items with the highest factor loadings were selected from all items that could be clearly assigned to a respective component (i.e. without cross-loading > 0.3 on other components). In a final revision, in order to avoid redundancies in the item wording, some items were exchanged with items of lower loadings but more relevant content. However, only two items could be allocated to the 6th component, as any possible 3rd item charged on at least one of the other five components. Therefore, the final item selection comprised of 15 items allocated to five components. These 15 items were again subjected to a PCA with five fixed components and Equamax-rotation to check whether the item reduction had an impact on the allocation of items to components (KMO = 0.899; Bartlett: *p* < .001; explained variance: 68.7%). Factor loadings on the respective components were high (range .656 to .853) and no item had loadings > 0.3 on more than one component (see Table 2).

**Table 2.** Final VASI-15 Items and factor loadings

Items	ePCA					CFA
	F1 (SR)	F2 (PE)	F3 (RE)	F4 (MV)	F5 (SE)	
If you live together with a mentally ill person, it is difficult to lead a life according to your own ideas.	**.853**	−.134	.090	.085	.164	**.801**
Living together with a mentally ill person restricts one’s own quality of life.	**.841**	−.178	.156	.086	.156	**.841**
In interacting with a person with mental illness, you invest a lot of energy and get only little in return.	**.656**	−.208	.176	.269	.172	**.673**
People with mental illness are a valuable addition to society.	−.155	**.805**	.031	−.040	−.117	**.672**
In general, I feel comfortable spending time with a person who is mentally ill.	−.195	**.774**	−.108	−.038	.005	**.642**
Interacting with mentally ill people can be very enriching for oneself.	−.090	**.743**	−.099	−.214	−.208	**.753**
It is damaging my reputation if a mental illness becomes known in my family.	.145	−.057	**.812**	.137	.062	**.613**
Having mentally ill people in the neighbourhood impairs the attractiveness of my residential area.	.125	−.087	**.765**	.197	.274	**.785**
Just like beggars, mentally ill people taint the appearance of the city.	.143	−.043	**.689**	.229	.287	**.732**
Going easy on people with mental illness in the workplace is unfair to those who do not have a mental illness.	.150	−.126	.102	**.788**	.076	**.618**
Mental illness is often only an excuse for laziness.	.091	−.030	.249	**.717**	.223	**.637**
Nowadays people who are mentally ill are shown too much consideration.	.149	−.142	.220	**.704**	.289	**.770**
Mentally ill people commit particularly cruel crimes.	.199	−.060	.100	.149	**.803**	**.679**
Mentally ill people represent a great danger for children.	.199	−.160	.278	.251	**.707**	**.795**
The neighbourhood should be warned about people with severe mental illness.	.135	−.165	.267	.193	**.705**	**.697**
Eigenvalue	5.574	0.908	1.716	0.949	1.158	
Explained Variance	14.285	13.315	13.901	13.326	13.873	

Annotations: factor loadings are shown for components of exploratory principal component analysis (ePCA) in subsample A (*N* = 2489) and of confirmatory factor analysis (CFA) in subsample B (*N* = 2494). Subscales: *SR* Self-realization, *PE* Personal Enrichment, *RE* Reputation, *MV* Meritocratic Values, *SE* Security, PCA initial Eigenvalues and explained variance after Equamax rotation are given

#### Confirmatory factor analysis (CFA)

**Fig. 2:**
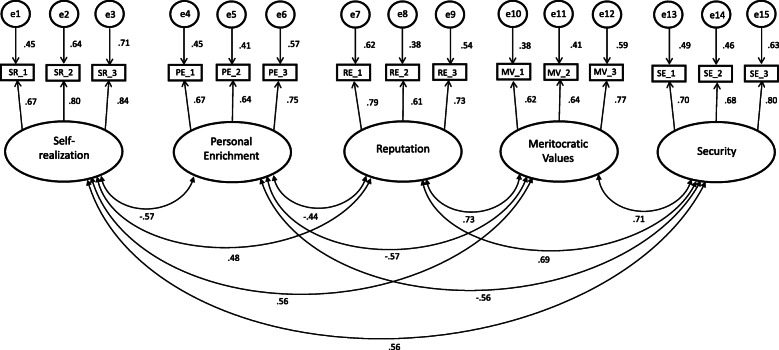
CFA of the five-factor model of the VASI-15

To verify the validity of the exploratory factor analysis (see Fig. 2), a CFA was performed on the 15 selected items using data from subsample B (*N* = 2494). A significant Mardia test (z = 58.244, *p* < .001) indicated the absence of a multivariate normal distribution, although skewness and excess of all items were below the limits proposed by West and colleges [36]. Despite the implementation of a Bollen-Stine-Bootstrap, the Chi^2^ test reached significance (X^2^(80) = 502.902; *p* = .001), which questioned the fit of the model. However, other goodness-of-fit indices consistently attested a good model fit (CFI = 0.969; TLI = 0.959; RMSEA = 0.046; SRMR = .0368). Factor loadings were again high for all items (range .614 to .842, see Table 2). A description of the VASI scales from a content perspective is given in the next section.

### VASI-15

#### Description of VASI-subscales and item analysis

Table 3 shows the 15 final VASI items, with their assignment to the five VASI subscales, which are described as follows:

**Table 3.** Item characteristics of the final 15 VASI-items

	VASI-item	Scale	Mean	Median	SD	γ_m_	ω	p_m_	r_it_
1	If you live together with a mentally ill person, it is difficult to lead a life according to your own ideas.	SR	3.29	3.0	1.108	−0.339	−0.505	0.659	0.533
2	Going easy on people with mental illness in the workplace is unfair to those who do not have a mental illness.	MV	2.34	2.0	1.146	0.489	−0.546	0.468	0.493
3^a^	People with mental illness are a valuable addition to society.	PE	2.72	3.0	1.040	0.073	−0.233	0.544	0.400
4	Mental illness is often only an excuse for laziness.	MV	1.76	1.0	1.019	1.234	0.777	0.353	0.510
5	Living together with a mentally ill person restricts one’s own quality of life.	SR	3.15	3.0	1.128	−0.187	−0.627	0.629	0.573
6	It is damaging to my reputation if a mental illness becomes known in my family.	RE	1.76	1.0	1.021	1.264	0.837	0.352	0.456
7^a^	Interacting with mentally ill people can be very enriching for oneself.	PE	3.36	3.0	1.079	−0.278	−0.345	0.671	0.522
8	Having mentally ill people in the neighborhood impairs the attractiveness of my residential area.	RE	1.71	1.0	0.977	1.370	1.316	0.342	0.572
9^a^	In general, I feel comfortable spending time with a person who is mentally ill.	PE	2.84	3.0	1.056	0.029	−0.311	0.567	0.414
10	In interacting with a person with mental illness, you invest a lot of energy and get only little in return.	SR	2.75	3.0	1.160	0.139	−0.738	0.550	0.591
11	Mentally ill people commit particularly cruel crimes.	SE	2.33	2.0	1.179	0.471	−0.641	0.467	0.539
12	Just like beggars, mentally ill people taint the appearance of a city.	RE	1.72	1.0	1.001	1.365	1.223	0.345	0.564
13	Nowadays people who are mentally ill are shown too much consideration.	MV	2.17	2.0	1.120	0.663	− 0340	0.434	0.613
14	The neighborhood should be warned about people with severe mental illness.	SE	2.29	2.0	1.198	0.525	−0.676	0.459	0.578
15	Mentally ill people represent a great danger for children.	SE	2.36	2.0	1.071	0.495	−0.404	0.452	0.645

Annotations: ^a^ inverse coded, *SD* standard deviation, *γ*_*m*_ skewness, *ω* kurtosis, *p*_*m*_ item difficulty, *r*_*it*_ corrected item-total correlations. Subscales: *SR* Self-Realization, *PE* Personal Enrichment, *RE* Reputation, *MV* Meritocratic Values, *SE* Security

Subscale 1 *Self-realization (SR)* focusses on the extent to which the presence of people with mental illness in the immediate environment is perceived as restricting one’s well-being and enjoyment of life as well as the ability to achieve one’s individual life goals.

Subscale 2 *Personal Enrichment (PE)* includes the extent to which dealing with mentally ill persons is perceived as an enrichment for one’s life and represents an openness to diversity. Underlying are humanistic values. In contrast to the other VASI scales, these values are not threatened but rather promoted by dealing with the mentally ill persons. Accordingly, the items of this scale are inversely coded and need to be inverted before they can be included in the calculation of an overall VASI score.

Subscale 3 *Reputation (RE)* deals with the loss of social status associated with mental illness. It addresses the extent to which being associated with mentally ill people would be considered detrimental to one’s reputation.

Subscale 4 *Meritocratic Values (MV)* deals with two aspects of individualistic values. First, an underlying belief that too much consideration is given to mentally ill people, which is perceived as unfair against those who are not mentally ill. Mentally ill people are perceived as receiving benefits for which the respondent feels more entitled. Second, the scale includes the attitude of personal responsibility in conjunction with a high level of self-improvement, according to the principle that everyone is responsible for themselves. Both aspects can be summarized under the label *Meritocratic Values*, where people achieve and earn what they deserve.

Subscale 5 *Security (SE)* deals with the perceived dangerousness of mentally ill people, to whom a propensity to violence and a willingness to commit particularly serious crimes are attributed. The attitudes recorded on this scale correspond most closely to the stigmatising attitudes associated with authoritarianism attitudes.

The VASI subscales *Self-realization* and *Security* relate to the similarly titled subscales of the basic values of Schwartz [16], whereas *Personal Enrichment* is comparable to Schwartz´ Self-Transcendence. *Reputation* and *Meritocratic Values* are factors which are not to be equated with the basic values of Schwartz in their importance.

Item characteristics of the 15 VASI items are shown in Table 3. The difficulty indices ranged from 0.34 to 0.67, thus attesting to a medium difficulty. The mean inter-item correlation was *r =* 0.326, thus attesting to an optimal item homogeneity. Corrected item-total correlations ranged from 0.40 to 0.65 and the internal consistency was good for the total VASI scale (Cronach’s α = 0.879) and acceptable for the five VASI subscales (SR: α = 0.805; PE: α = 0.729; RE: α = 0.756; MV: α = 0.721; SE: α = 0.769). Mean Scores of the VASI scales separated by gender and age-groups are shown in Table 4. The final VASI questionnaire and notes on its evaluation can be found in Additional file 1.

**Table 4.** VASI-15 Mean Scores

	N	VASI-15 Total Score	Self-Realization (SR)	Personal Enrichment (PE)	Reputation (RE)	Meritocratic Values (MV)	Security (SE)
Males							
≤ 24 years	288	7.26 (±1.935)	8.73 (±2.749)	8.49 (±2.598)	5.34 (±2.466)	6.48 (±2.690)	7.24 (±2.715)
25–34 years	479	7.52 (±2.140)	8.72 (±2.969)	8.60 (±2.407)	5.99 (±3.109)	6.68 (±2.985)	7.62 (±3.053)
35–44 years	445	7.70 (±2.109)	9.27 (±2.962)	9.18 (±2.546)	5.93 (±2.826)	6.66 (±2.935)	7.46 (±2.975)
45–54 years	549	7.52 (±2.061)	9.37 (±2.894)	9.37 (±2.717)	5.44 (±2.435)	6.41 (±2.636)	7.02 (±2.897)
55–64 years	521	7.23 (±1.950)	9.10 (±2.962)	9.16 (±2.470)	5.23 (±2.404)	6.03 (±2.594)	6.62 (±2.773)
≥ 65 years	235	7.30 (±1.978)	9.15 (±2.717)	9.28 (±2.448)	5.23 (±2.269)	6.40 (±2.424)	6.42 (±2.837)
All age-groups	2517	7.44 (±2.046)	9.08 (±2.911)	9.04 (±2.559)	5.55 (±2.646)	6.44 (±2.747)	7.10 (±2.917)
Females							
≤ 24 years	258	6.59 (±1.561)	8.30 (±2.518)	8.12 (±2.406)	4.48 (±1.897)	5.57 (±2.223)	6.46 (±2.355)
25–34 years	447	7.29 (±1.931)	8.76 (±2.987)	9.14 (±2.626)	5.15 (±2.559)	6.24 (±2.794)	7.18 (±2.782)
35–44 years	437	7.65 (±2.099)	9.48 (±3.096)	9.35 (±2.778)	5.32 (±2.545)	6.65 (±2.781)	7.48 (±2.975)
45–54 years	528	7.36 (±2.018)	9.16 (±2.994)	9.12 (±2.532)	4.98 (±2.497)	6.58 (±2.736)	7.01 (±3.031)
55–64 years	531	7.29 (±2.033)	9.29 (±2.997)	9.37 (±2.625)	4.78 (±2.195)	6.11 (±2.515)	6.89 (±2.915)
≥ 65 years	257	7.33 (±1.678)	9.44 (±2.840)	9.57 (±2.387)	4.63 (±1.781)	6.24 (±2.322)	6.78 (±2.601)
All age-groups	2458	7.30 (±1.962)	9.11 (±2.953)	9.16 (±2.613)	4.94 (±2.344)	6.29 (±2.635)	7.02 (±2.855)
Total Sample							
≤ 24 years	546	6.94 (±1.797)	8.52 (±2.648)	8.31 (±2.513)	4.94 (±2.255)	6.05 (±2.519)	6.88 (±2.578)
25–34 years	928	7.41 (±2.043)	8.74 (±2.973)	8.86 (±2.526)	5.57 (±2.884)	6.46 (±2.902)	7.40 (±2.932)
35–44 years	883	7.67 (±2.106)	9.36 (±2.986)	9.26 (±2.668)	5.62 (±2.705)	6.65 (±2.857)	7.46 (±2.974)
45–54 years	1078	7.45 (±2.041)	9.27 (±2.944)	9.24 (±2.630)	5.21 (±2.476)	6.49 (±2.686)	7.01 (±2.962)
55–64 years	1054	7.26 (±1.991)	9.19 (±2.978)	9.26 (±2.551)	5.00 (±2.310)	6.07 (±2.554)	6.75 (±2.846)
≥ 65 years	492	7.32 (±1.825)	9.30 (±2.783)	9.43 (±2.418)	4.92 (±2.048)	6.32 (±2.370)	6.61 (±2.719)
All age-groups	4981	7.37 (±2.006)	9.09 (±2.931)	9.09 (±2.587)	5.25 (±2.519)	6.36 (±2.693)	7.06 (±2.886)

#### Discriminant & convergent validity

Correlations of the VASI-scores and other scales are depicted in Table 5. As expected, the highest correlations were found between the overall VASI score and the individual VASI subscales (*R =* .636 to .787). Intercorrelations between the five VASI-subscales were of medium size, with highest correlations between subscale *Security* and subscale *Meritocratic Values* (*R =* .522) as well as with subscale *Reputation* (*R =* .518) and lowest correlations between subscale *Personal Enrichment* and subscales *Reputation* (*R =* .307), *Meritocratic Values* (*R =* .355) and *Security* (*R =* .367).

**Table 5.** Rank-Correlation coefficients (Spearman) between VASI scores and instruments assessing stigmatization and personal values

		N	VASI-15 Total Score	Self-Realization (SR)	Personal Enrichment (PE)	Reputation (RE)	Meritocratic Values (MV)	Security (SE)
Scale Inter-correlation	VASI-15 Total Score	4983	**1**	.738***	−.636***	.718***	.751***	.787***
	Subscale Self-Realization	4983	.738***	**1**	−.419***	.400***	.405***	.453***
	Subscale Personal Enrichment	4983	−.636***	−.419***	**−1**	−.307***	−.355***	−.367***
	Subscale Reputation	4983	.718***	.400***	−.307***	**1**	.507***	.518***
	Subscale Meritocratic Values	4983	.751***	.405***	−.355***	.507***	**1**	.522***
	Subscale Security	4983	.787***	.453***	−.367***	.518***	.522***	**1**
Convergent validity	SDS: Mean Score	3984	.676***	.455***	−.582***	.450***	.463***	.575***
	SSMI: Aware Score	3986	.120***	.105***	.022	.088***	.090***	.162***
	SSMI: Agree Score	3986	.670***	.421***	−.343***	.506***	.547***	.625***
	KSA-3: Authoritarianism Score	4983	.334***	.214***	−.190***	.231***	.297***	.296***
	PVQ: Self-Enhancement Score	2494	.129***	.052**	−.063**	.152***	.121***	.111***
	PVQ: Self-Transcendence Score	2491	−.412***	−.194***	.318***	−.379***	−.387***	−.283***
	PVQ: Conservation Score	2494	.071***	.078***	−.074***	−.009	.021	.099***
	PVQ: Openness Score	2493	−.102***	−.094***	.136***	−.082***	−.038	−.036
Discriminant validity	KSE-G: positive qualities Score	4981	−.120***	−.062***	.119***	−.147***	−.085***	−.054***
	KSE-G: negative qualities Score	4982	.182***	.093***	−.022	.218***	.186***	.151***
	KSE-G: Social Desirability Score	4980	−.192***	−.100***	.075***	−.228***	−.175***	−.136***

Annotations: * *p* < .050; ** *p* < .010; *** *p* < .001; coefficients > |.200| are depicted in bold font

Concerning associations with other stigma related scales, a strong positive correlation between the VASI total score and the SDS total score was shown (*R =* .676). Of the VASI subscales, the strongest correlations with social distance were found for *Security* (*R =* .575) and *Personal Enrichment* (*R =* −.582). The two sub-scales of the SSMI show very different association patterns with the VASI. Strong positive correlations were found for the SSMI-subscale *Stereotype Agreement*, which measures individual endorsement of the common public stereotypes, and the VASI total score (*R =* .670) as well as the VASI-subscale *Security* (*R =* .625) and as well as moderate associations for the other VASI-subscales. Neither the VASI total score, nor any VASI-subscale showed considerable correlations (R > .02) for the SSMI subscale *Stigma Awareness*, which assesses whether people know existing stereotypes about mental illnesses.

Associations between VASI scores and value-specific scales were in most cases significant but of smaller magnitude. Small positive correlations were found between the VASI total score (*R =* .334) and all VASI subscales (*R =* .190 to .297). We observed a recurring pattern across all VASI subscales mainly regarding two values. The Self-Transcendence scale (PVQ) showed a negative correlation with all VASI subscales, whereas the Authoritarianism scale (KSA-3) showed an overall positive correlation (the inversely coded Personal Enrichment subscale showing opposite relations). Authoritarianism was most strongly related to the VASI subscales *Security* and *Meritocratic Values*.

Associations with the KSE-G scores assessing social desirability were also significant, although arguably only due to the large sample size, as most correlations coefficients were well below 0.2. Only the VASI subscale *Reputation* showed small correlations with the KSE-G social desirability score (*R =* −.228). Looking at the two KSE-G subscales, one can assume that this correlation is mainly based on an association with the subscale *Minimizing negative properties* (*R =* .218).

## Discussion

This study reports the development and the validity of the Value-based Stigma Inventory (VASI). It is the first questionnaire measuring mental illness stigma referring to a value-based concept. In addition to conservative/authoritarian value attitudes, an attempt was made to measure stigmatization in connection with liberal/modern values.

During the VASI development a preliminary pool of 68 items was generated by a mental health stigma expert team and a person with lived experience. These were surveyed with established stigma and value scales via an online panel. One strength is the questionnaire development by means of a large sample (*N* = 4983), which allows to capture the various value orientations prevalent among the German population. For the subscale development, an exploratory factor analysis (EFA) was carried out within one half of the sample, in which five factors were identified. Taking factor loadings and item contents into account, three items per factor were selected. This model could be verified by a confirmatory factor analysis (CFA) within the other half of the sample. The Bollen-Stine corrected Chi^2^ test did reach significance, thus questioning the good model fit, however, the chi-square value is known to be susceptible to large sample sizes [32, 37], therefore it cannot be excluded that the statistical significance is mainly due to the large N of our sample. Accordingly, other goodness-of-fit indices consistently met the required cutoffs [33–35], thus attesting a good model fit.

A five-factor structure of value-based stigma is proposed, forming the subscales *Self-Realization, Personal Enrichment, Reputation*, *Meritocratic Values and Security,* which are medium to high interrelated and represent a spectrum from liberal/modern to conservative/authoritarian stigma-associated values. Alternatively to this recommended calculation of 5 subscale scores, a total score can also be calculated (see Additional file 1).

The validity of the new instrument was investigated using established scales for the assessment of mental health stigma (SDS; SSMI), personal values (PVQ-21; KSA-3), as well as social desirability (KSE-G). The findings suggest a good convergent validity, with medium to high correlation coefficients between the VASI subscales and the SDS- and SSMI_Agree_-scores corresponding to our assumptions. At the VASI subscale level the correlation coefficients in both stigma scales are highest in *Security* and lowest in *Personal Enrichment*. As could be expected, people who are more likely to feel threatened and endangered by people with mental illness tend to have a greater need for social distance, while people who experience dealing with the mentally ill as enriching are more open for social contacts. In accordance with the recommendations by Cohen [15], we found correlations between the VASI scales and value scales to be of medium range. Correlations of the VASI subscales with the value scales approve that *Security* is associated with an authoritarian value orientation and *Personal Enrichment* is related to values of self-transcendence. This indicates that people with authoritarian values are stigmatizing to a higher degree than others, whereas values of self-transcendence function as a protective factor against stigmatization. This trend appears constantly on all VASI subscales. Contrary to our assumptions, the subscales do not significantly differ in the correlations with different values. This indication for the existence of a single stigma relevant value dimension (authoritarian values vs. self-transcendence) should nevertheless be viewed critically. It can be assumed that people with liberal values are more in conflict with their values (tolerance vs. individualism) than people with authoritarian values. Therefore, they may quickly see through the liberal stigma items and tend to respond in terms of social desirability. This could be considered in more subtle item formulations or the use of implicit measurement methods [38] in the future. Furthermore, a response shift due to an assumed higher education level among participants with a liberal basic attitude is possible.

An expansion of the liberal/modern concept of values, which was viewed primarily in connection with openness and tolerance, with the aspect of individualism should be considered as a possibility based on the data and should be further investigated in the future. The factor *Self-Realization* reflect aspects of personal development and self-optimization, which are associated with stigmatization of people with mental illness when presenting as a potential threat to relevant individualistic values. These results point to a field of tension between an anti-stigmatizing attitude and liberal stigmatization. For example, Wetherell and coworkers [39] found that both conservatives and liberals discriminate against ideologically divergent groups if those violate their core values. Thereby abstract values can mitigate (universalism, egalitarianism) or reinforce (traditionalism) discrimination. This suggests that discrimination takes place among liberal-minded people, but in contrast to conservative persons, it might be mitigated by their liberal values.

Concerning discriminatory validity, low correlations to the subscale SSMI_*Aware*_ are shown. In distinction to SSMI_*Agree*_ it is not measuring stigma per se [40], but rather the awareness of stereotype in the public. The VASI asks for agreement with stereotypical opinions of mental illness, therefore the differences in correlations between the two SSMI subscales are as to be expected. The correlation with the social desirability scale are within an acceptable small range except for the subscale *Reputation* and the KSE-G subscale *Negative Qualities*. It can be argued that people who hide own negative characteristics in order to appear socially desirable are likely to associate mental illness with loss of reputation and prestige.

With the VASI scale, we introduce a valid instrument that integrates public stigma of mental health with individual value preferences. The scale includes items pertaining to stigma relevant values beyond authoritarianism, as well as to stigma protective values. This extension of the content of the stigma assessment by a multifaceted value concept is an important strength of this scale and distinguishes it from other already established stigma scales.

### Limitations and perspectives

Although the sample of this study is still comparatively large, there are two limitations inherent in data collection via online panel. First, the sample cannot be regarded as representative of the German-speaking resident population, as no statistical random selection was carried out. Participants with older age and a higher education were overrepresented. However, it can be assumed that the size of the sample ensures that people with the most diverse attitudes and values were included. Second, a relatively high percentage of survey participants (18.2%) were not included because they answered the survey below the pre-determined minimum response time of 11 min. When determining the time limit, we were guided by the processing times of independent consultants with questionnaire experience, the distribution of the pretest processing times and the recommendations of a visual inspection of the frequency distribution of response times by Wise and DeMars [41]. Although this time limit was intended to exclude participants with rapid guessing behavior, nevertheless an exclusion of participants with solution behavior but a high processing time cannot be excluded.

Reliability was determined by calculating the internal consistencies. However, since we conducted a one-time, anonymous online survey, no information on the test-retest reliability of the VASI can be derived from this study. The investigation of retest reliability needs to be considered in future use of the instrument.

## Conclusions

In total the study offers sufficient data to substantiate the validity of the VASI. The questionnaire showed a clear factor structure and convergent and discriminant validity. The use of the VASI can be suggested in further studies to measure public stigma of mental illness including personal stigma relevant value orientations. The VASI forms an interface between psychiatric, psychological and sociological fields of research. One possible field of application could be the detection of stigma in different socio-economic milieus in order to identify different patterns of discrimination against persons with mental illness.

## Supplementary Information

**Additional file 1.** Value-based Stigma Inventory (VASI).

## Data Availability

The datasets obtained and/or analysed during the current study are available from the corresponding author on reasonable request.

## Abbreviations

CFA: Confirmatory Factor Analysis
CFI: Comparative Fit Index
EFA: Exploratory Factor Analysis
ESS: European Social Survey
EV: Eigenvalues
KMO: Kaiser-Meyer-Olkin
KSA-3: Authoritarianism Short Scale
KSE-G: Social Desirability–Gamma Short Scale
PCA: Principal Component Analysis
PPMI: Prejudice against People with Mental Illness Scale
PVQ: Portrait Values Questionnaire
RMSEA: Root Mean Square Error of Approximation
SDS: Social Distance Scale
SRMR: Standardized Root Mean Residual
SSMI: Self-Stigma in People with Mental Illness scale
TLI: Tucker-Lewis Index
VASI: Value-based Stigma Inventory
WMM: What Matters Most
